# Selective induction of alternatively spliced FynT isoform by TNF facilitates persistent inflammatory responses in astrocytes

**DOI:** 10.1038/srep43651

**Published:** 2017-03-07

**Authors:** Chingli Lee, Clara Y. B. Low, Siew Ying Wong, Mitchell K. P. Lai, Michelle G. K. Tan

**Affiliations:** 1 Department of Clinical Translational Research, Singapore General Hospital, The Academia, Level 9, Discovery Tower, 20 College Road, 169856, Singapore; 2 Department of Pharmacology, Yong Loo Lin School of Medicine, National University of Singapore, Unit 09-01, Centre for Translational Medicine (MD6), 14 Medical Drive, Kent Ridge 117599, Singapore

## Abstract

Fyn tyrosine kinase has been implicated in the pathogenesis of Alzheimer’s disease (AD). We have previously reported that upregulation of the FynT isoform in AD brains was partly associated with astrocyte activation. In this study, we demonstrated selective FynT induction in murine cortex and primary astrocyte culture after prolonged exposure to inflammatory stimulants, suggesting that FynT may mediate persistent neuroinflammation. To delineate the functional role of astrocytic FynT in association with TNF-mediated inflammatory responses, immortalized normal human astrocytes (iNHA) stably expressing FynT kinase constitutively active (FynT-CA) or kinase dead (FynT-KD) mutants were treated with TNF and compared for inflammatory responses using high-throughput real-time RT-PCR and Luminex multi-analyte immunoassays. FynT-CA but not FynT-KD mutant exhibited drastic induction of proinflammatory cytokines and chemokines after prolonged exposure to TNF, which could be attenuated by treating with Fyn kinase inhibitor PP2 or silencing via FynT-specific DsiRNA. FynT kinase activity-dependent induction of PKCδ expression, PKCδ phosphorylation, as well as NFκB activation was detected at the late phase but not the early phase of TNF signaling. In conclusion, selective FynT induction by TNF may facilitate persistent inflammatory responses in astrocytes, which is highly relevant to chronic neuroinflammation in neurodegenerative diseases including but not limited to AD.

Neuroinflammation driven by activation of brain-resident immune cells, namely microglia and astrocytes, is a hallmark associated with neurodegenerative conditions, including Alzheimer’s disease (AD). In AD, amyloid beta (Aβ) has been recognized to initiate an inflammatory response through microglia activation and astrocyte recruitment^1^,^2^. Furthermore, several studies have demonstrated that tumour necrosis factor (TNF), which is known to stimulate glial response in mediating induction and secretion of inflammatory mediators^3^, has also been found to be elevated in the serum, cerebrospinal fluid and cortex of AD patients^4^,^5^, as well as in Aβ-treated microglia and astrocytes^6^,^7^. In contrast to the well-recognized immunocompetent nature of microglia, astrocytes are better known for their neurosupportive roles in the brain, and more recently, research has highlighted critical roles played by astrocytes in the prolonged neuroinflammatory process in AD^8^.

The non-receptor tyrosine kinase, Fyn, has recently been implicated in the pathogenesis of AD, which is mainly based on its neuropathogenic role that links Aβ toxicity to tau pathology^9^,^10^. In fact, Fyn is better known for its role in immune system functions and has been studied extensively in mediating T cell development and activation^11^, mast cell degranulation^12^, as well as oligodendrocyte myelination^13^. The diverse biological functions of Fyn are likely associated with the splicing of two mutually exclusive exons which generate the brain-predominant FynB and the hematopoietic cells-predominant FynT isoform^14^,^15^. Functional studies revealed that FynT is more efficient than FynB in mediating calcium mobilization^16^ and stimulating lymphokine secretion^17^. Furthermore, selective upregulation of FynT was detected in activated T cells under phorbol ester treatment^18^, whereas elevated FynB observed in virally infected T cells could attenuate activation^19^. Both observations support the notion that stimuli-mediated alternative splicing of Fyn may modulate and fine-tune T cell activation. Here, we demonstrate that the modulation of alternative splicing of Fyn may not be limited to controlling T cell activation, but also regulate neuroinflammatory responses. Indeed, we previously reported that selective upregulation of FynT detected in post-mortem AD brains was partially associated with reactive astrogliosis^20^. Furthermore, astrocytes under Aβ treatment^20^, as well as TNF treatment as demonstrated in the current study, both led to selective upregulation of FynT expression, suggesting that the modulation of alternative splicing of Fyn in favoring FynT expression may be associated with astrocyte-mediated neuroinflammatory responses.

Compelling evidence of Fyn being able to modulate the production of proinflammatory cytokines has been mainly reported in peripheral immune cells including but not limited to activated mast cells^21^,^22^ and natural killer cells^23^,^24^. The involvement of Fyn in neuroinflammation has not been studied as extensively. However, it has been reported that fibrillar Aβ initiated signaling that led to proinflammatory response was dependent on Fyn kinase in microglia^25^. Along the same line, a double transgenic Fyn/hAPP model demonstrated increase of reactive astrocytosis as compared to singly transgenic mice expressing hAPP or Fyn alone at the age of 6–7 months^26^, suggesting that Fyn may potentiate astrocyte activation during the disease progression. A recent study further demonstrated that TNF and lipopolysaccharide (LPS)-treated microglia exhibited rapid Fyn activation for downstream inflammatory response, as well as Fyn induction after prolonged treatment^27^. Most importantly, inhibiting Fyn activity by treating AD mice with AZD0530 restored their spatial memory deficits, which was not solely limited to rescue synapse loss and tau phosphorylation but also reduce microglia activation^28^. Taken together, Fyn has been shown to modulate neuroinflammation, likely through activation of microglia and astrocytes. However, no study has attempted to delineate the isoform-specific role of Fyn to this aspect.

In this study, we identified that FynT being the specific isoform modulated by inflammatory stimulants *in vivo* and in primary astrocyte culture and speculate that astrocytic FynT may mediate persistent neuroinflammation through TNF signaling pathway, and contribute to the progression of AD. Using recombinant approaches, we focused on studying the underlying mechanism of FynT kinase activity in association with TNF-mediated inflammatory responses in astrocytes.

## Results

### FynT expression consistently correlated with astrocytic markers in primary rat mixed cortical culture

**Figure 1:**
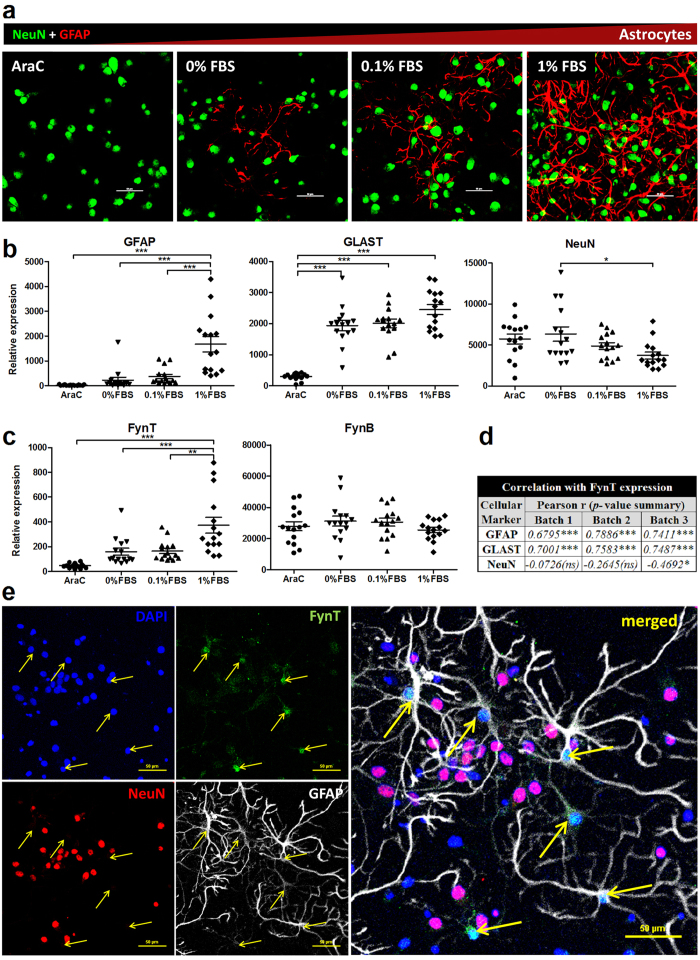
FynT expression consistently correlated with astrocytic markers in primary rat mixed cortical culture. Three independent batches of primary rat mixed cortical culture were maintained and analyzed on 7–10 DIV under different culture conditions (n = 15 in each group): 1 μM AraC in serum free, or no AraC with increasing serum concentration (0, 0.1 and 1% FBS). (**a**) Representative double-IF staining of GFAP (red) and NeuN (green) were used to monitor astrocytic and neuronal population, respectively. Scale bars = 50 μm. (**b**) Expression of GFAP and GLAST astrocytic marker and NeuN neuronal marker and (**c**) expression of FynT and FynB isoform were monitored by real-time RT-PCR. Horizontal lines with error bars in dot plots indicate mean ± SEM. Significantly different was determined by one-way ANOVA with Bonferroni’s *post*-*hoc* test (*P ≤ 0.05, **P ≤ 0.01, ***P ≤ *0.001*). (**d**) Significant correlation between the expression of FynT and each cellular markers were indicated by Pearson’s correlation coefficients (*r*) and p-value summary (*P ≤ 0.05, **P ≤ 0.01, ***P ≤ *0.001*, (*ns*) not significant). (**e**) Triple-IF staining of FynT (green), NeuN (red) and GFAP (white) was performed on primary rat mixed cortical culture with DAPI nuclear stain (blue). Yellow arrows indicated astrocytes that positively stained with FynT. Scale bars = 50 μm.

We have reported that higher proportions of FynT expression was detected in primary astrocytes as compared to primary neurons^20^. To further corroborate our previous observation, primary mixed culture derived from rat embryonic cortical tissues were maintained in 1 μM Cytosine β-D-arabinofuranoside (AraC) mitotic inhibitor (to inhibit astrocyte propagation) or increasing fetal bovine serum (FBS) concentrations (0.1 and 1%, to promote astrocyte propagation) and compared for FynT and FynB isoform expression. Representative double-immunofluorescence (IF) staining of the primary rat mixed cortical cultures revealed that Glia Fibrillary Acidic Protein (GFAP)-positive astrocytic population was absent under AraC treatment but increased with higher FBS concentration (Fig. 1a, red). In contrast, no obvious change in the number of NeuN-positive neuronal cells was detected under the same conditions (Fig. 1a, green). In line with IF results, real-time RT-PCR data showed that expression of GFAP and GLAST astrocytic markers correspondingly increased with higher FBS concentration (Fig. 1b). On the other hand, expression of NeuN transcripts (also known as RBFOX3) seemed slightly decreased under 1% FBS culture (Fig. 1b), probably due to increased astrocytic population resulting in relatively lower percentage of neuronal cells in the mixed culture.

Interestingly, a corresponding increase in FynT expression was detected in mixed cultures maintained in FBS, with higher expression in 1% compared to 0.1% FBS, while FynB expression remained unchanged under all culture conditions (Fig. 1c). Correlation analyses further confirmed that FynT consistently correlated with GFAP and GLAST in all three batches of culture (Fig. 1d). We thus conclude that FynT is predominantly expressed in astrocytes, whereas FynB may be expressed at a similar level in both astrocytes and neurons.

In accordance with our findings, triple-IF staining of FynT (Fig. 1e, green), NeuN (Fig. 1e, red) and GFAP (Fig. 1e, white) on primary rat mixed cortical cultures confirmed that FynT immunoreactivities were predominantly detected in GFAP-positive astrocytes (Fig. 1e, yellow arrows) but not in NeuN-positive neurons.

### Selective FynT induction in murine cortex and primary astrocyte culture after prolonged exposure to inflammatory stimulants

**Figure 2:**
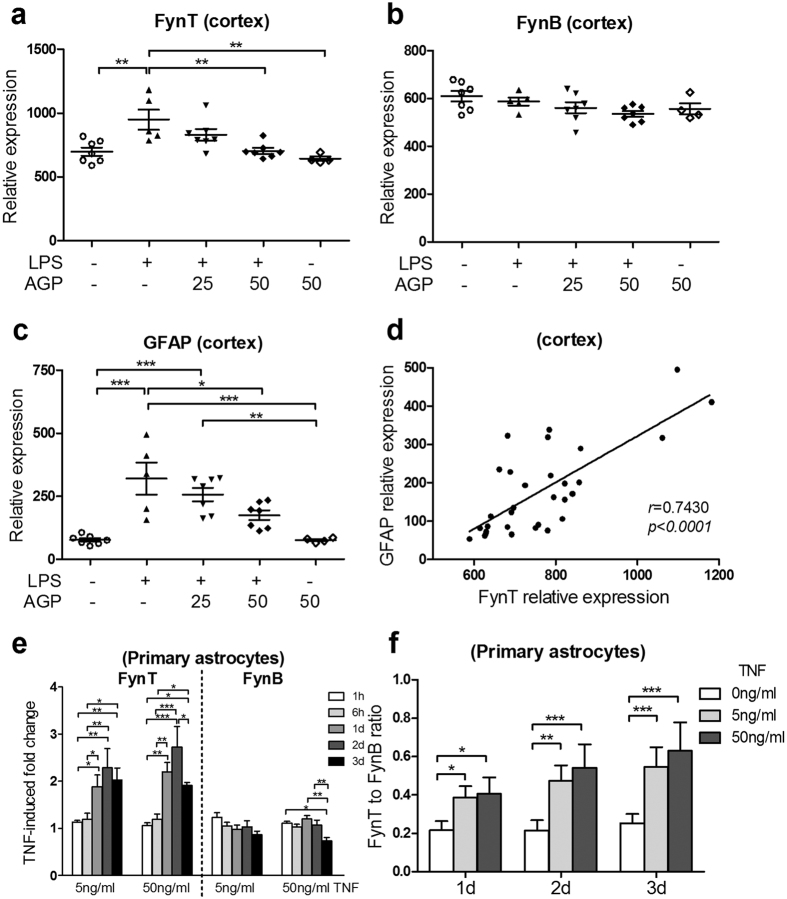
Selective FynT induction in murine cortex and primary astrocyte culture after prolonged exposure to inflammatory stimulants. An *in vivo* murine neuroinflammation model was set up by three intraperitoneal injection of LPS or PBS as vehicle control over 24 h, and one hour after each injection, the mice were given oral gavages of either AGP (25 or 50 mg/kg) to attenuate inflammatory response or PEG as vehicle control and sacrificed four hours after the last oral gavage. Real-time RT-PCR was used to monitor the expression of (**a**) FynT, (**b**) FynB and (**c**) GFAP in the mouse cortex in each treated group (n = 4 to 7 per group) which were normalized to GAPDH. (**d**) Significant correlation between FynT and GFAP expression in the mouse cortex was determined by Pearson’s correlation coefficient. (**e**) Primary astrocyte culture treated with 5 or 50 ng/ml TNF or vehicle control were monitored for FynT and FynB expression at 1 h, 6 h, 1d, 2d and 3d post-treatment by real-time RT-PCR. The TNF-induced fold change was calculated by setting the normalized relative signal intensity of corresponding non-treated control at each time point as 1. Values are mean ± SEM of three independent experiments. (**f**) FynT to FynB ratio was determined by RT-PCR using a pair of common primers spanning the alternative spliced exon of Fyn to amplify both FynB and FynT which differed in 9 base pairs followed by fragment analysis. Data are mean ± SEM of three independent experiments. Significant differences were determined using two-way ANOVA with Bonferroni’s *post*-*hoc* test (*P ≤ 0.05, **P ≤ 0.01, ***P ≤ *0.001)*.

A recent study demonstrated that prolonged treatment of TNF or LPS led to Fyn induction in microglia^27^. Here, we adopted a widely-used systemic LPS-induced murine neuroinflammation model^29^, which we have recently reported that an anti-inflammatory agent, andrographolide (AGP) was able to attenuate the induced inflammatory responses in the mouse cortex^30^. Remarkably, FynT, but not FynB expression was significantly increased in the murine cortex after systemic LPS administration (Fig. 2a and b). Furthermore, AGP attenuated LPS-induced neuroinflammatory response was accompanied by the reduction of FynT expression in a dose-dependent manner, with no impact on FynB expression (Fig. 2a and b). Consistently, LPS-induced GFAP expression could also be attenuated by AGP treatment (Fig. 2c). GFAP expression was significantly and positively correlated with FynT expression (Fig. 2d), supporting a link between FynT induction and astrogliosis in association with neuroinflammation.

Primary astrocyte culture treated with 5 or 50 ng/ml TNF were monitored closely for the changes of FynT and FynB expression. Consistently, we detected significant increment of TNF-induced fold change of FynT at day 1 post-treatment which could be sustained till day 3 (Fig. 2e). In contrast, significant reduction of FynB expression was detected at day 3 post-treatment of 50 ng/ml TNF (Fig. 2e). An independent approach of determining FynT to FynB ratio further confirmed that higher FynT proportion was detected in primary astrocytes after prolonged exposed to TNF (Fig. 2f), suggesting that selective FynT induction may be partially contributed by the activation of alternative splicing machinery in favor of FynT expression. Based on these observations, we hypothesize that FynT kinase activity may have an as yet undefined role in modulating proinflammatory responses in astrocytes after prolonged exposure to TNF.

### FynT kinase activity facilitated the induction of proinflammatory cytokine expression after prolonged exposure to TNF

**Figure 3:**
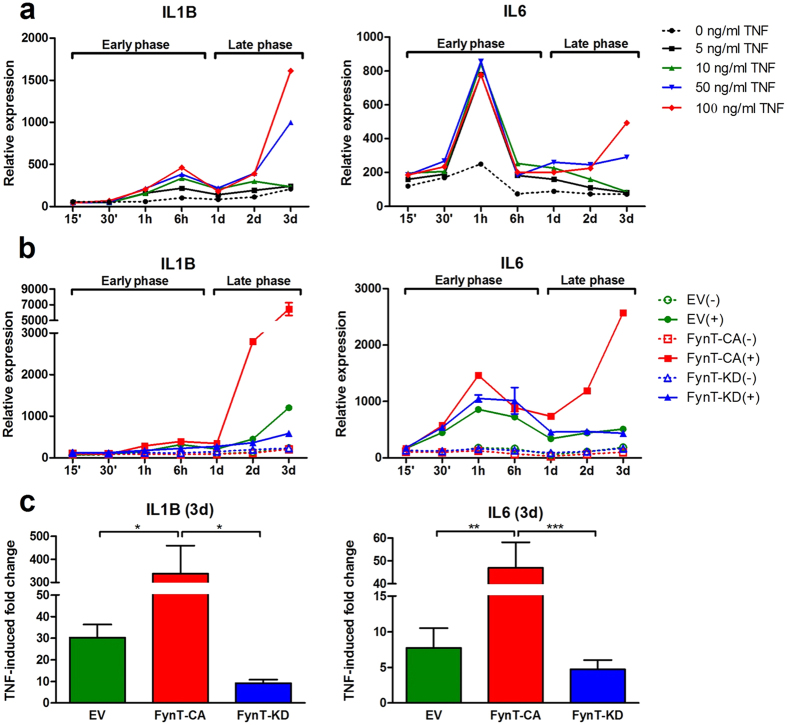
FynT kinase activity facilitated the induction of pro-inflammatory cytokines expression after prolonged exposure to TNF. (**a**) Parental iNHA treated with 0, 5, 10, 50 and 100 ng/ml TNF were monitored for IL1B and IL6 mRNA expression at different time points (15 min, 30 min, 1 h, 6 h, 1d, 2d and 3d) using real-time RT-PCR. Relative expressions of genes were normalized by geometric means of housekeeping genes (see Methods). Biphasic induction was determined by the detection of a peak at the early phase (within minutes to hours) followed by a second increment at the late phase (from 1d onwards). (**b**) iNHA clones stably expressing EV (green), FynT-CA (red) or FynT-KD (blue) were treated with 50 ng/ml TNF (+, solid line) or left untreated (−, dotted line) and monitored for IL1B and IL6 mRNA expression at different time points using real-time RT-PCR. Relative expressions of genes were normalized by the geometric means of housekeeping genes, setting the expression level of untreated control of each clone at time 0 as 100. This was a representative data of two independent studies. (**c**) TNF-induced fold changes of IL1B and IL6 in EV, FynT-CA and FynT-KD clone at day 3 post-treatment were calculated by dividing the expression under 50 ng/ml TNF treatment to untreated control as determined using real-time RT-PCR. Values are the means ± SEM of seven independent experiments. Significantly different was determined by one-way ANOVA with Bonferroni’s *post*-*hoc* test (*P ≤ 0.05, **P ≤ 0.01, ***P ≤ *0.001*).

Figure 3a shows that immortalized normal human astrocytes (iNHA) exhibited a rapid induction of interleukin 1β (IL1B) and interleukin 6 (IL6) at the early phase, within minutes to a few hours of exposure to TNF. Interestingly, prolonged exposure (≥1 days) to higher doses of TNF (50 and 100 ng/ml) led to a second cytokine induction, with the highest increment at day 3 (Fig. 3a).

To determine the role of FynT kinase activity in modulating TNF-mediated proinflammatory responses, we generated iNHA clones with ectopic expression of FynT wild-type (FynT-WT) and FynT mutants (constitutively active FynT-CA or kinase dead FynT-KD), as well as empty vector control (EV). Immunoblot analysis confirmed ectopic expression of FynT as well as their corresponding kinase activities of each clone using anti-Fyn and anti-pTyr416 antibodies (Suppl. Fig. S1).

Upon 50 ng/ml TNF treatment, all iNHA clones demonstrated rapid induction of IL1B and IL6 expression at the early phase, regardless of their ectopic FynT kinase activity (Fig. 3b). Remarkably, only FynT-CA mutant clone demonstrated predominant induction of IL1B and IL6 at the late phase, with the highest increment at day 3, whereas EV and FynT-KD clones exhibited minimal or basal induction of IL1B and IL6 (Fig. 3b), suggesting that FynT kinase activity may facilitate the induction of proinflammatory cytokines after prolonged exposure to TNF.

We further measured the induction fold change of cytokines in each iNHA clone after exposure to TNF for 3 days in seven independent treatments. FynT-CA clone exhibited the highest fold change induction for IL1B (mean ± SEM = 338.2 ± 121.3) and IL6 (46.9 ± 11.2) (Fig. 3c). Although it is not statistically significant, we observed that a slight attenuation of TNF-induced fold change in FynT-KD clone as compared to EV clones for IL1B (9.1 ± 1.7 versus 30.3 ± 6.0) and IL6 (4.7 ± 1.3 versus 7.7 ± 2.8) (Fig. 3c).

### FynT kinase activity-dependent modulation of differential expression and secretion of inflammatory-associated markers after prolonged exposure to TNF

**Figure 4:**
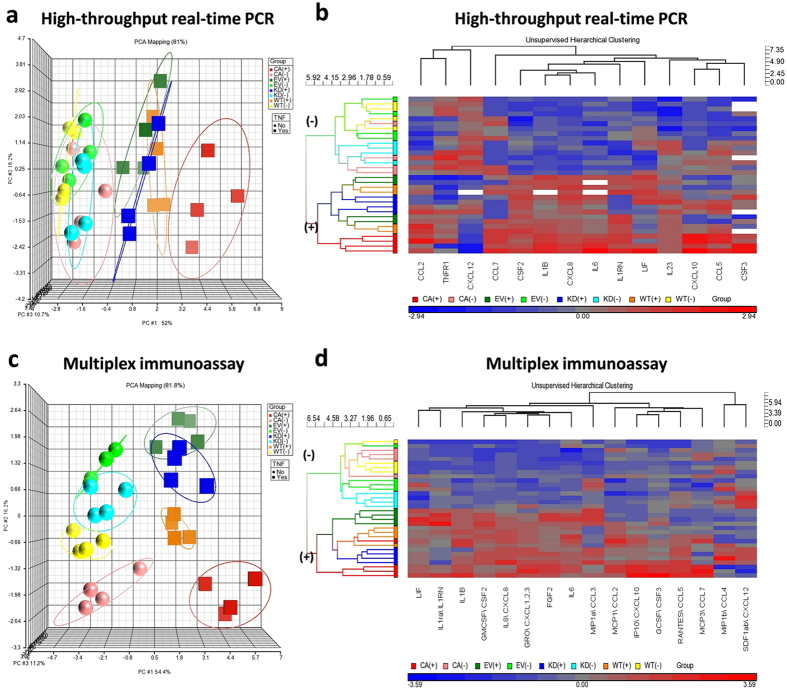
FynT kinase activity-dependent modulation of differential expression and secretion of inflammatory-associated markers after prolonged exposure to TNF. iNHA stable clones with ectopic expression of FynT with different kinase activity (WT, CA and KD), as well as empty vector control (EV) were treated with 50 ng/ml TNF (+) or left untreated (−) for 3 days in four independent experiments. The expression profile of a panel of 14 inflammatory associated markers (CCL2, CCL5, CCL7, CXCL8, CXCL10, CXCL12, CSF2, CSF3, IL1B, IL1RN, IL6, IL23, LIF, TNFR1) were determined using Biomark HD high-throughput real-time RT-PCR system and data presented in (**a**) and (**b**). Furthermore, the secretion profile of a panel of 16 inflammatory-associated markers in the media was determined using MILLIPLEX^®^ Multiplex Assays with Luminex platform and data presented in (**c**) and (**d**). Identified batch effect was removed before processing with subsequent analysis using PCA and hierarchical clustering using Partek Genomic Suite. (**a**,**c**) PCA identified the first 3 principal components (PCs) that explained 81% of the variation in the dataset. Each object represents an iNHA stable clone, either TNF treated clones (cubes) or untreated clones (sphere). The variation of four biological replicates in each clone could be visualized by the size of each ellipse. TNF-treated clones (cubes) were well-segregated from untreated clones (spheres), with TNF-treated FynT-CA (red cubes) showing the greatest distance from the others. (**b**,**d**) Two-dimensional unsupervised hierarchical clustering analysis for segregation of iNHA clones based on their characteristics in response to TNF treatment. The gene expression levels of the inflammatory-associated markers were shown with the columns (genes) and rows (samples) in cluster order. The relative value to the median among all samples are shown in colour with a continuum of expression levels from dark blue (lowest) to bright red (highest) and missing data are shown in white. Corresponding gene names are listed at bottom. The left dendrogram lists the clones studied and provides a measure of the relatedness of expression in each sample.

To reinforce the observation that FynT tyrosine kinase activity facilitated TNF-induced proinflammatory cytokines expression after prolonged incubation, EV control, FynT mutants of FynT-CA and FynT-KD as well as FynT-WT clones were treated with TNF or left untreated for 3 days in four independent experiments and subjected to expression profiling from a panel of 14 inflammatory associated markers using Biomark HD system’s high-throughput real-time RT-PCR. These markers could be broadly categorized into proinflammatory chemokines (CCL2, CCL5, CCL7, CXCL8, CXCL10, CXCL12), proinflammatory cytokines (CSF2, CSF3, IL1B, IL6, IL23), anti-inflammatory cytokines (IL1RN, LIF) and TNF receptor, TNFR1. Using Principal component analysis (PCA), the variability of TNF-induced inflammatory markers expression in each iNHA clone could be clearly visualized in a 3D scatter plot, allowing the clones with distinct expression profiles to be identified easily. In Fig. 4a, the PCA 3D scatter plot was shown to account for 81% of the variation in the dataset. Most of the untreated clones (spheres) were clustered together and well separated from TNF-treated clones (cubes), suggesting a general TNF-induced differential expression of inflammatory markers. Furthermore, we observed that upon TNF treatment, FynT-CA clone (red cubes) exhibited the greatest distance from the other clones (Fig. 4a).

Consistent with the PCA results, unsupervised hierarchical clustering analysis of 14 inflammatory markers revealed that iNHA clones could be well segregated into two independent branches, as untreated (−) and TNF-treated (+) group on the left dendrogram (Fig. 4b). FynT-CA exhibited the highest induction of inflammation-associated markers in response to TNF treatment, which was mainly marked as bright red in the majority of the heat map column, and clustered as an independent sub-branch, highlighted in red in the left dendrogram (Fig. 4b).

To confirm that FynT kinase activity regulates the secretion of proinflammatory cytokines and chemokines after prolonged exposure to TNF, conditioned media were collected from iNHA clones at day 3 post-treatment and measured for 16 inflammatory-associated markers using multiplex immunoassays (MILLIPLEX^®^). PCA 3D scatter plot displayed 81.8% of the variation in the dataset (Fig. 4c), showing clear segregation of untreated clones (spheres) from treated clones (cubes). Furthermore, TNF-treated FynT-CA clones (red cubes) exhibited the greatest distance from the others and closer to FynT-WT clones (orange cubes), whereas FynT-KD clones (blue cubes) were closer to the EV clone (Fig. 4c). Consistent with the PCA results, unsupervised hierarchical clustering analysis revealed that FynT-CA could be clustered as an independent sub-branch with cytokine secretion pattern closer to that of FynT-WT (Fig. 4d). Our findings indicate that FynT kinase activity may positively modulate TNF-induced inflammatory responses.

### Attenuation of TNF-induced proinflammatory cytokines by inhibiting FynT kinase activity or by specific silencing of ectopic FynT expression

**Figure 5:**
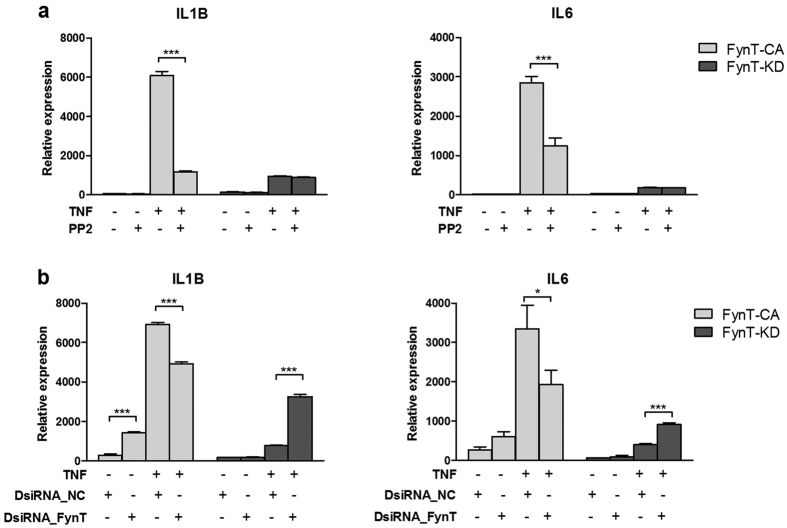
Attenuation of TNF-induced proinflammatory cytokines by inhibiting FynT kinase activity or by specific silencing of ectopic FynT expression. (**a**) Inhibition of FynT kinase activity in FynT-CA and FynT-KD clone was carried out by 6 h pre-incubation of 10 μM PP2 or DMSO control vehicle, followed by 3 days co-treatment with or without 50 ng/ml TNF and monitored for IL1B and IL6 expression using real-time RT-PCR. (**b**) Specific silencing of FynT iNHA FynT-CA and FynT-KD clones were carried out by transfecting with 30 nM FynT-specific DsiRNA mixture (DsiRNA_FynT) or 30 nM Negative Control duplex (DsiRNA_NC) followed by 3 days co-treatment with or without 50 ng/ml TNF and monitored for IL1B and IL6 expression using real-time RT-PCR. Relative expressions of genes were normalized by geometric means of housekeeping genes. Values are the means ± SEM of three independent biological replicates. Two-way ANOVA with Bonferroni’s *post*-*hoc* test was conducted separately in each clone to monitor the impact of PP2 and DsiRNA on TNF-induced IL1B and IL6 expression (*P ≤ 0.05, **P ≤ 0.01, ***P ≤ *0.001*).

Selective Src family kinases (SFK) inhibitor PP2 was able to inhibit ectopic FynT kinase activity in FynT-CA clone in a dose-dependent manner, as determined by reduction of pTyr416 immunoreactivities without affecting Fyn protein level (Suppl. Fig. S2). We demonstrated that PP2 treatment significantly attenuated the induction of IL1B and IL6 by TNF in FynT-CA clone (Fig. 5a), which confirmed that TNF-induced proinflammatory cytokine at the late phase was dependent on FynT kinase activity. On the other hand, we observed no impact of PP2 treatment on TNF-induced IL1B and IL6 expression in FynT-KD clone (Fig. 5a), suggesting that other SFK members may not be involved in modulating basal induction of IL1B and IL6 at the late phase of TNF treatment.

Using Dicer-substrate siRNAs (DsiRNA) randomized sequence duplex (DsiRNA_NC) as control, we demonstrated that treatment FynT-CA and FynT-KD clones with pooled DsiRNAs specific targeting FynT (DsiRNA_FynT) (duplex sequences can be found in Suppl. Fig. S3a) resulted in ≥90% inhibitory efficiency in FynT expression, with no inhibitory effect on FynB expression under treatment with or without TNF (Suppl. Fig. S3b). Specific silencing of ectopic FynT expression in FynT-CA clone significantly attenuated TNF-induced IL1B and IL6 expression (Fig. 5b). Note that the attenuation of TNF-induced IL1B and IL6 expression in FynT-CA clone by DsiRNA silencing was less effective than PP2 treatment. Conversely, specific silencing of the ectopic dominant negative FynT expression in FynT-KD demonstrated significant enhancement of TNF-induced IL1B and IL6 expression, although the overall expression remain relatively low as compared to FynT-CA clone (Fig. 5b).

### FynT kinase activity-dependent induction of PKCδ expression and phosphorylation after prolonged exposure to TNF

**Figure 6:**
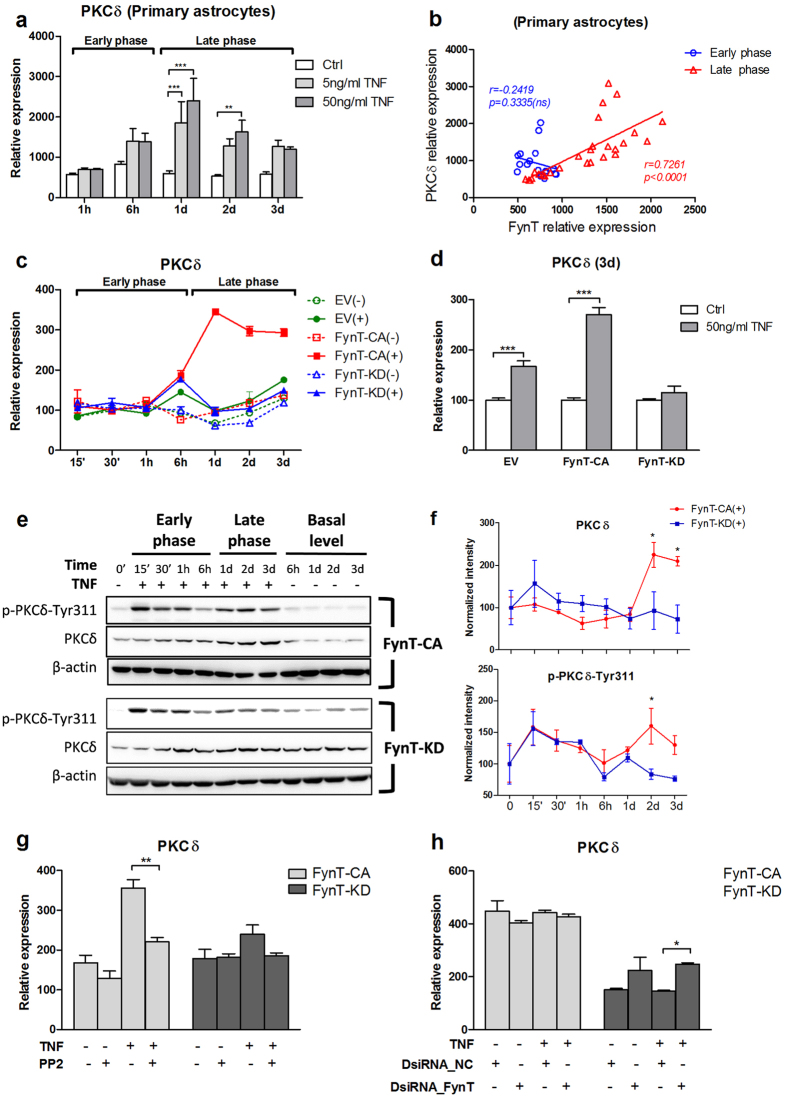
FynT kinase activity-dependent induction of PKCδ expression and phosphorylation after prolonged exposure to TNF. Primary rat astrocytes were treated with or without 5 or 50 ng/ml of TNF for 1 h, 6 h, 1d, 2d and 3d in three biological replicates and monitored for (**a**) PKCδ expression by real-time RT-PCR and determined (**b**) correlation between PKCδ and FynT expression independently at early phase (1 h, 6 h) and late phase (1d, 2d and 3d) by Pearson’s correlation coefficient. (**c**) EV, FynT-CA and FynT-KD clone were treated with 50 ng/ml TNF (+) or left untreated (−) and collected at different time points. Relative expression of PKCδ were determined by real-time RT-PCR, after normalization by geometric means of housekeeping genes and set the untreated control at time 0 as 100. This was a representative data of two independent studies. (**d**) TNF-induced PKCδ expression at day 3 post-treatment was performed in five biological replicates. (**e**) Representative immunoblot of PKCδ protein level and phospho-PKCδ-Tyr311 in FynT-CA and FynT-KD clones treated with or without 50 ng/ml TNF treatment at the early and late phase were shown. β-actin was used as a loading control. Full-length blots are presented in Suppl. Fig. S4. (**f**) Quantification of PKCδ and p-PKCδ-Tyr311 immunoblots were carried out by normalization to β-actin, and set the untreated control at time 0 as 100. Graph showed means ± SEM of three to four independent experiments. (**e**) 10 μM PP2 or DMSO control vehicle treated FynT-CA and FynT-KD clone with or without 50 ng/ml TNF treatment were monitored for PKCδ expression in three biological replicates. (**f**) FynT-CA and FynT-KD were transfected with DsiRNA_FynT or DsiRNA_NC with or without 50 ng/ml TNF treatment were monitored for PKCδ expression in three biological replicates. Two-way ANOVA with Bonferroni’s *post*-*hoc* test was conducted for (**a**), (**d**), (**f**) and each clone in (**g**) and (**h**) to monitor the impacts of FynT kinase activity on TNF-induced PKCδ expression (*P ≤ 0.05, **P ≤ 0.01, ***P ≤ *0.001*).

Fyn to PKCδ (also known as PRKCD) signaling axis has been shown to mediate proinflammatory responses under LPS and TNF treatment in microglia^27^. In this study, we observed that primary rat astrocytes exhibited induction of PKCδ transcripts at the late phase of TNF treatment (Fig. 6a). Positively correlation between FynT and PKCδ expression was exclusively detected at the late phase (Fig. 6b, red, Pearson correlation coefficient *r* = *0.7261, p* < *0.0001*), suggesting that TNF-induced PKCδ expression may be relevant to FynT induction at the late phase. Likewise, after prolonged exposure to TNF, only FynT-CA clone exhibited PKCδ induction at the late phase (Fig. 6c). Further analysis on day 3 of five independent batches of TNF treatment revealed that TNF-induced PKCδ expression was dependent on FynT kinase activity, showing augmentation in FynT-CA clone and inhibition in TNF-KD clone as compared to EV control (Fig. 6d).

Consistently, we observed that PKCδ protein level was upregulated at the late phase in FynT-CA but not in FynT-KD clone, which was accompanied by the increased phospho-Tyr311, a marker of PKCδ activation (Fig. 6e and f). Furthermore, inhibition of Fyn kinase activity by PP2 significantly attenuated TNF-induced PKCδ expression in FynT-CA clone (Fig. 6g), confirming that PKCδ induction was dependent on FynT kinase activity. However, we noticed that while transfecting with DsiRNA_NC scramble control, FynT-CA clone did not respond to TNF stimulation for PKCδ induction suggesting that the transfection procedure alone could have modulated PKCδ expression in FynT-CA and thus confounded the effect of FynT silencing (Fig. 6h). On the other hand, specific silencing of the ectopic dominant negative FynT expression in FynT-KD demonstrated significant enhancement of TNF-induced PKCδ expression (Fig. 6h).

### FynT kinase activity facilitated NFκB activation at the late phase of TNF signaling

**Figure 7:**
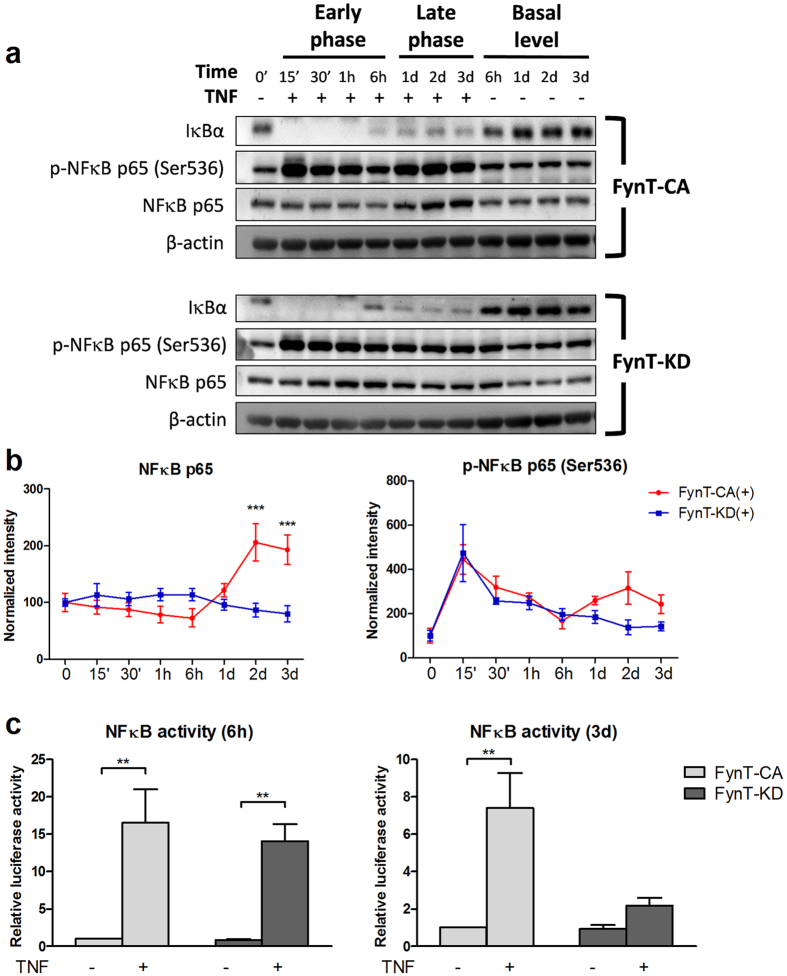
FynT kinase activity facilitated NFκB activation at the late phase of TNF signaling. FynT-CA and FynT-KD were treated with 50 ng/ml TNF (+) or left untreated (−) and monitored for activation of NFκB at the early and late phase. (**a**) Representative immunoblot of key molecules involved in NFκB pathway and β-actin as a loading control was shown. Full-length blots are presented in Suppl. Fig. S4. (**b**) Quantification of NFκB p65 and phospho-NFκB p65 (Ser536) immunoblots were carried out by normalization to β-actin and set the untreated control at time 0 as 100. Graph showed means ± SEM of four independent experiments. (**c**) NFκB reporter assay was compared between FynT-CA and KD clones at the early time point (6 h) and late time point (3d). NFκB activity were determined after normalizing against Renilla luciferase activity and set the value of untreated FynT-CA as 1. Values are the means and ± SEM of six independent biological repeats. Two-way ANOVA with Bonferroni’s *post*-*hoc* test was conducted for (**b**) and (**c**) to monitor the impact of FynT kinase activity on TNF-induced NFκB activation (**P ≤ *0.01*).

Paracrine stimulation by TNF can lead to activation of a key inflammatory regulator, Nuclear factor-kappa B (NFκB) and result in the expression of multiple proinflammatory cytokines and chemokines^31^,^32^. Immunoblot analyses revealed that increase of phospho-NFκB p65 (Ser536) immunoreactivities was accompanied by the rapid degradation of IκBα protein in both FynT-CA and FynT-KD clones shortly after TNF stimulation (Fig. 7a). This was consistent with the NFκB reporter assay results, demonstrating comparable TNF-induced NFκB luciferase activity in both FynT-CA and FynT-KD clones at 6 h post-treatment (Fig. 7c, left panel), suggesting that FynT kinase activity has no impact in modulating TNF-induced NFκB signaling pathway at the early phase. Instead, TNF-induced elevation of NFκB p65 protein level and its phosphorylation at Ser536 were specifically detected in FynT-CA clone one day after treatment and remained high at day 3 post-treatment, in contrast to no obvious changes in FynT-KD clones (Fig. 7a and b). Consistently, we observed that FynT-CA clone exhibited significantly higher NFκB activity after 3 days prolonged incubation with TNF, in contrast to much lower NFκB activity in the FynT-KD clone (Fig. 7c, right panel), suggesting that FynT kinase activity may modulate NFκB signaling after prolonged incubation with TNF and subsequently lead to induction of proinflammatory cytokine expression.

## Discussion

### Selective FynT induction after prolonged exposure to inflammatory stimulants: a possible modulation of alternative splicing of relevance to chronic neuroinflammation

The biological relevance of astrocytes exposure to prolonged TNF treatment would be in the context of chronic inflammation where TNF constitutively activated NFκB and persistently induced inflammatory responses in neurodegenerative diseases, including but not limited to AD^33^. It has been reported that the induction of early, intermediate and late response genes under TNF treatment are highly associated with the temporal oscillation pattern of NFκB activation, with the late response genes being activated after persistent stimulation by higher dose of TNF^34^,^35^. Panicker *et al*. demonstrated that microglial Fyn expression could be augmented by prolonged exposure to TNF and LPS, suggesting that Fyn is likely a late response gene^27^. Here, we observed isoform-specific induction of FynT in mouse cortex and primary astrocyte culture after prolonged exposure to inflammatory stimulants (Fig. 2). Interestingly, being an anti-inflammatory agent, AGP attenuated LPS-induced neuroinflammatory response was accompanied by the significant reduction of LPS-induced FynT expression (Fig. 2a), suggesting a close relationship between FynT induction and inflammatory response. These findings are in line with our previous report demonstrating Aβ selectively induced astrocytic FynT expression in mixed cortical culture, as well as increased FynT immunoreactivity detected in reactive astrocytes in AD^20^, suggesting that astrocytes may be responsive to different types of stimuli in triggering FynT induction and subsequently modulating downstream inflammatory responses. However, the underlying mechanisms of isoform-specific induction of Fyn by TNF remain unclear, and probably involve the interplay between transcription activation, alternative splicing and mRNA stability.

Alternative splicing has been proposed to regulate and fine-tune the diversity and plasticity required for immune system^36^ and nervous system^37^. However, the modulation of alternative splicing under different stimuli is still poorly defined. It was reported that Fyn exhibited an alteration in isoform expression in response to T cell stimulation, as demonstrated by phorbol ester PMA-induced splicing that favored FynT expression^18^. Herein, we postulate that alternative splicing machinery may also be modulated in astrocytes in favor of FynT expression in response to TNF and Aβ stimuli based on our findings. Sequence homology to activation-responsive sequence (ARS) has been found in FynB-specific exon (also known as exon 7A of Fyn)^18^. However, whether selective FynT induction is due to activation-dependent repression of FynB requires further confirmation.

### FynT kinase activity facilitated inflammatory responses at the late phase of TNF treatment

Consistent with the observation of increased astrocytic FynT expression after prolonged TNF treatment (Fig. 2e and f), we identified that FynT kinase activity-dependent induction of proinflammatory cytokines at the late phase, as demonstrated by prominent induction in FynT-CA clone contrasting with the significantly less responsive FynT-KD clone (Fig. 3). We have performed prolonged treatment of TNF in more than one independent clone and obtained similar findings with greater TNF-induced fold change of IL1B and IL6 in FynT-CA clones as compared to FynT-KD clones (data not shown). Furthermore, inhibiting FynT kinase activity and specific silencing of ectopic FynT in FynT-CA clone, both significantly attenuated TNF-induced proinflammatory cytokines (Fig. 5), confirming that FynT kinase activity indeed plays a main role in facilitating inflammatory responses after prolonged exposure to TNF. Interestingly, we also observed a ‘rescue’ phenotype after knocking down the dominant negative FynT mutant in FynT-KD clone, which appeared to improve the responsiveness to TNF (Fig. 5b). Taken together, our findings supported that FynT kinase activity may facilitate TNF-induced prolonged inflammatory responses. Hence, inhibiting FynT kinase activity could be a potential therapeutic strategy in alleviating chronic inflammation in neurodegenerative diseases. Indeed, inhibiting Fyn activity by AZD0530 has demonstrated significant reduction of microglia activation in AD mice^28^, supporting the role of Fyn, and probably FynT specifically, in modulating neuroinflammation in AD.

### FynT kinase activity mediated TNF-signaling pathway through PKCδ and NFκB activation

SFKs have been shown to modulate TNF-induced NFκB activation through phosphorylating certain signaling molecules. For example, c-src phosphorylates IkBα^38^,^39^ and Fyn phosphorylates PKCδ at Tyr-311^27^. Among several potential mechanisms that linked PKCδ kinase activity to enhanced NFκB activation, phosphorylation of NFκB p65 at Ser536 by PKCδ has been shown to induce its DNA binding affinity and transactivation leading to proinflammatory cytokine expression^40^. In order to understand the impact of FynT kinase activity on TNF-signaling pathway, we monitored closely the time course of phosphorylation of PKCδ-Tyr311 and NFκB-Ser536 in FynT-CA and FynT-KD clones treated with TNF.

Both FynT-CA and FynT-KD clones exhibited rapid phosphorylation of PKCδ-Tyr311 (Fig. 6e and f) and NFκB p65-Ser536 (Fig. 7a and b), suggesting that FynT kinase activity has no major impact on TNF signaling at the early phase. Interestingly, a second induction of phosphorylation of PKCδ-Tyr311 and NFκB p65-Ser536 could only be detected in FynT-CA clone at the late phase, which was accompanied by the significant increase of PKCδ and NFκB p65 protein levels (Figs 6f and 7b, respectively). The results are consistent with NFκB reporter assay data showing significant induction of NFκB activity in FynT-CA but not FynT-KD at day 3 of post TNF treatment (Fig. 7c). These observations explain why overt induction of inflammatory markers was detected in FynT-CA clone but not in FynT-KD clone (Fig. 4), as many inflammatory markers are target genes of NFκB signaling. The significance of our findings would be that FynT kinase activity could potentially propagate astrocyte-mediated inflammatory responses in the presence of TNF.

In addition to PKCδ phosphorylation at Tyr-311, FynT kinase activity may also be directly or indirectly involved in transcriptional regulation of PKCδ after prolonged exposure to TNF (Fig. 6c and d). This speculation was further confirmed by the attenuation of TNF-induced PKCδ expression in FynT-CA clone after inhibiting FynT kinase activity by PP2 treatment (Fig. 6g). However, specific silencing FynT by DsiRNAs failed to attenuate PKCδ transcripts in FynT-CA clone (Fig. 6h). Further analyses revealed that upon transfection with DsiRNA_NC scramble control, FynT-CA clone was unresponsive to TNF stimulation for PKCδ induction, suggesting that the transfection procedure alone may modulate PKCδ expression to elevate basal level in FynT-CA and thus confounded the effect of FynT silencing in attenuating PKCδ expression (Fig. 6h). Concomitantly, we observed basal level of IL1B and IL6 increased by DsiRNA_NC transfection, suggesting that PKCδ may closely modulate the expression of inflammatory cytokines including IL1B and IL6 (Fig. 5b). On the other hand, specific silencing of the ectopic dominant negative FynT expression in FynT-KD demonstrated significant enhancement of TNF-induced PKCδ expression (Fig. 6h), which was accompanied by elevation of IL1B and IL6 (Fig. 5b). In summary, we concluded that FynT kinase activity facilitates TNF-induced inflammatory responses which correspond well with PKCδ and NFκB phosphorylation and activation at the late phase, likely through non-canonical signaling pathways.

In summary, we have demonstrated that primary astrocytes exhibited selective FynT induction under prolonged treatment of TNF, which is similar to our previous observation under Aβ treatment^20^. Although the underlying mechanisms of the isoform-specific induction have not been defined, our findings evidently point to FynT being a mediator of sustained inflammatory processes in astrocytes which can potentially aggravate chronic inflammation in neurodegenerative diseases. Our findings, taken together with the established role of Fyn in modulating proinflammatory cytokines in immune cells highlights to us that FynT could be a potential therapeutic target for intervention of chronic inflammatory responses in neurodegenerative diseases including but not limited to AD.

## Methods

### Primary mixed cortical and astrocyte culture

Primary cortical mixed cultures and astrocyte cultures were established from embryonic (E18) and postnatal (P0 to P1) Sprague Dawley rat brains, respectively, after protocol approval from the SingHealth Institutional Animal Care and Use Committee (2014/SHS/950). All procedures were carried out in accordance with the approved guidelines and regulations. Briefly, cortices were dissected and meninges were removed in ice cold Hank’s balanced salt solution. To obtain primary cortical mixed culture, dissected cortices were digested using papain dissociation system (Worthington Biochemical Corporation), centrifuged and resuspended in Neurobasal (NB) medium supplemented with 2.5% B27, 0.25% GlutaMAX™, 1× penicillin-streptomycin, with or without 0.1 or 1% FBS. 1 × 10^6^ cells were seeded onto wells coated with 0.1 mg/ml Poly-D-lysine hydrobromide (Sigma) and cultured at 37 °C in a humidified 5% CO_2_ incubator. Half-change of media was done at day 4 *in vitro* (DIV 4). Neuron-enriched cultures were maintained in 1 μM AraC (Sigma) without FBS from DIV 4 onwards. On DIV 7, culture medium was replaced with NB medium minus phenol red with the same supplements for each corresponding conditions and treated with TNF.

To obtain primary astrocyte culture, isolated cortices were digested with 0.05% trypsin/0.5 mM EDTA in 1× PBS, filtered through Falcon 40 mm nylon cell strainer, centrifuged and resuspended in DMEM-F12 supplemented with 10% FBS, 1 mM L-Glutamine and 1× Penicillin-Streptomycin. Cells were plated at 1 × 10^7^ cells per 75 cm^2^ flask and kept in 5% CO_2_ incubator at 37 °C with media changed every 2 days. The confluent glial culture (12–14 DIV) was agitated at 250 rpm for 24 h on an orbital shaker with media changed twice to remove microglia. The adherent astrocytes were trypsinized and re-plated at a density of 5 × 10^5^ cells per well in 6-well plates in 0.5% FBS containing DMEM-F12 and treated with TNF.

### *In vivo* model

The systemic LPS-induced murine neuroinflammation model that we adopted in current approach has been reported in a separate study by our group^30^. Briefly, ICR male mice were subjected to three intraperitoneal injection of LPS (3 mg/kg per injection) or PBS as vehicle control at time 0, 6 h and 24 h, and one hour after each LPS injection, the mice were given oral gavages of 25 or 50 mg/kg andrographolide (AGP) or polyethylene glycol (PEG) as vehicle control. The mice were sacrificed four hour after the last oral gavage. Brain tissues were harvested for RNA isolation using TRIzol reagent (Invitrogen).

### FynT stably expressing iNHA clones

iNHA were primary human astrocytes stably transfected with constructs encoding E6, E7 and human telomerase reverse transcriptase (hTERT)^41^. iNHA cells were maintained in DMEM High Glucose supplemented with 10% FBS and 1× MEM non-Essential Amino Acids. pCMV6 Entry vector carrying the open reading frame of FynT-WT with C-terminal tagged of Myc-FLAG (OriGene Technologies) was subjected to site-directed mutagenesis to generate the constitutively active (CA) (FynT-Y528F, with an A/T switch at position 1583 resulting in a mutation from tyrosine to phenylalanine at amino acid position 528); or the kinase dead (KD) (FynT-K296M, with an A/T switch at position 887 resulting in a mutation from lysine to methionine at amino acid position 296). iNHA were transfected with FynT-WT, CA or KD mutants, or pCMV6 vector control (EV), and selected for individual clones in media containing 600 μg/ml of Geneticin.

### Double and triple-IF staining

Primary cortical cells grown on coverslips were fixed in 3.7% formaldehyde in PBS for 10 min, rinsed with PBS, permeabilized with 0.1% Triton X-100 in PBS for 5 min, rinsed with PBS, blocked with 10% normal goat serum in PBS at room temperature for 1 h. Cells were co-incubated overnight with two different species of primary antibodies at 4 °C, followed by washing and incubating with corresponding secondary antibodies at room temperature for 1 h. For triple-IF, cells were followed by staining with fluorophore-conjugated antibody. Coverslips were mounted in Vectashield mounting medium containing DAPI. Images were visualized and captured using the Nikon A1R confocal microscopy system. Antibodies in used are listed in Suppl. Table S1.

### RNA isolation, reverse transcription, real-time RT-PCR and fragment analysis

RNA extraction was performed using TRIzol reagent (Invitrogen) coupled with column purification. The aqueous phase obtained after centrifugation of TRIzol-Chloroform mixture was mixed with same volume of 70% ethanol and purified using NucleoSpin^®^ RNA kit (Macherey-Nagel) according to manufacturer’s instruction. 1 μg of the isolated total RNA was reverse-transcribed (RT) using High-Capacity cDNA RT kit (Applied Biosystems) in accordance to the manufacturer’s protocol. Amplification of the 2-times diluted cDNA was carried out in GoTaq^®^ qPCR Master Mix (Promega) containing 200 nM primer sets (Suppl. Table S2) using the 7500 Fast Real-Time PCR Machine (Applied Biosystems) with program setting: 50 °C, 2 min; 95 °C, 2 min; followed by 40 cycles of 95 °C, 15 sec and 60 °C, 1 min. Standard curves were generated by 10× serial dilution of template DNA for each gene to calculate relative signal intensity of each sample. Normalization was performed in each sample by dividing the relative signal intensity of gene of interest to geometric mean of β-actin, GAPDH and 18 S rRNA. To determine FynT to FynB ratio in rat cDNA, 25 cycles of RT-PCR was performed using a pair of common primers spanning the alternative spliced exon of Fyn. Fragment analysis of FynT and FynB PCR product was then performed using capillary electrophoresis as described previously^20^. Primers in used are listed in Suppl. Table S2.

### High Throughput Real-Time PCR

Specific target amplification (STA) was carried out for cDNA in 1× TaqMan PreAmp Master Mix (Applied Biosystem) containing 0.05 μM pooled target primer pairs, under the following condition: 95 °C for 2 min; 95 °C, 15 sec for 10 cycles; 60 °C, 4 min. After a clean-up step by treating with Exonuclease I (ExoI), samples were diluted 10-fold in TE buffer. Preparation and loading of samples and assay mix onto 192.24 Dynamic Array integrated fluidic circuit (IFC) was performed according to the manufacturer’s instructions. Briefly, 1.8μl of each diluted ExoI-treated STA product was mixed with 2 μl of 2× SsoFast EvaGreen Supermix with low ROX (Bio-Rad) and 0.2 μl of Delta Gene Sample Reagent (Fluidigm) before applied to IFC. 0.4 μl of each primer set (50 μM) was mixed independently with 2 μl of 2× Assay loading reagent (Fluidigm) and 1.6 μl of 1× DNA suspension buffer (10 mM Tris, 0.1 mM EDTA, pH 8.0) as assay mix before applied to IFC. After completion the mixing of samples and assays in IFC Controller RX, IFC was then transferred to BioMark HD system (Fluidigm) and ran with program setting: 95 °C, 1 min; followed by 30 cycles of 96 °C, 5 sec and 60 °C, 20 sec. The melting curve analysis consisted of 3 sec at 60 °C followed by heating up to 95 °C with a ramp rate of 1 °C/3 sec. Standard curves of each gene was generated by 10× serial dilution of a pooled template DNAs for calculating relative signal intensity of each sample. Data normalization was carried out similar to real-time RT-PCR as mentioned above.

### Multiplex immunoassays

Conditioned culture media were collected at day 3 post TNF-treatment, centrifuged for 10 min at 4 °C to remove cell debris and stored at −80 °C until use. Measurement of released cytokines and chemokines including CCL2(also known as MCP1), CCL3(MIP1α), CCL4(MIP1β), CCL5(RANTES), CCL7(MCP3), CXCL1,2,3(GRO), CXCL8(IL8), CXCL10(IP10), CXCL12(SDF1α+β), CSF2(GMCSF), CSF3(GCSF), FGF2, IL1RN(IL1ra), IL1B(IL1β), IL6, LIF were by MILLIPLEX^®^ Multiplex Assays with Luminex LX200 instrument (Millipore) according to the manufacturer’s instruction. Analysis was performed using MILLIPLEX^®^ Analyst Software Version 3.4 (Millipore).

### Gene silencing and treatment

Three DsiRNAs specific targeting FynT (DsiRNA_FynT), as well as normal control duplex (DsiRNA_NC), were purchased from Integrated DNA Technologies (sequences found in Suppl. Fig. S4a). iNHA clones were seeded at 2–3 × 10^5^ cells per well and transfected the following day with DsiRNA using HilyMax transfection reagent (Dojindo Laboratories). Briefly, 9 μl of HilyMax reagent was added to 120 μl Opti-MEM^®^ I Reduced-Serum Medium (GIBCO) containing 3 μl of either 20 μM pooled DsiRNA_FynT or DsiRNA_NC, mixed well and incubated for 15 min at room temperature. The mixture was then added directly into each well. A second transfection was required to be performed two day after the initial transfection to effectively knockdown the ectopic FynT expression in each iNHA clone.

### NFκB reporter assay

iNHA clones were co-transfected with NFkB(1) luciferase Reporter Vector (pNFκB-luc) (Panomics) and Renilla luciferase control reporter vector (pRL-CMV) (Promega) at a ratio of 100:1. At 24 h post-transfection, cells were left untreated or treated with 50 ng/ml TNF for 6 hours or 3 days and harvested for luciferase activity measurement using Dual-Luciferase^®^ Reporter Assay Kit (Promega) according to manufacturer’s instructions. NFκB transcriptional activity was determined by Firefly luciferase activities after normalizing for transfection efficiency against Renilla luciferase activities.

### Immunoblot analysis

Samples were lysed directly in 2× Laemmli Sample Buffer (Bio-Rad) with addition of 5% of β-mercaptoethanol and boiled for 5 min. Lysates were measured for protein amount using the 2-D Quant Kit (GE Healthcare) according to manufacturer’s instructions. After sample separation by 10% SDS-PAGE and transferred onto nitrocellulose membranes using the iBlot^®^ Dry Blotting System (Invitrogen), the membranes were blocked with Bløk-PO Noise Cancelling Reagents (Millipore) for 1 h, and incubated overnight with primary antibodies (Suppl. Table S1) in blocking solution at 4 °C. After rinsing with Tris-Buffered Saline with 0.1% Tween 20 (TBST), the membrane was incubated with corresponding secondary antibodies conjugated with horseradish peroxidase in blocking solution for 1 h at room temperature, washed with TBST and developed using Immobilon Western Chemiluminescent HRP Substrate (Millipore). Chemiluminescence signals were captured by UVIchemi image analyser (UVItec). Reprobing was performed using Restore^TM^ western blot stripping buffer (Pierce) at room temperature for 20 min and rinsed with TBST.

### Graphs and statistical analyses

Partek^®^ Genomics Suite Version 6.6 software was used to perform Principal component analysis (PCA) and unsupervised hierarchical clustering analysis. All data were transformed to log2 base scale and checked for batch effects. Once a batch effect was identified, it will be removed using a proprietary function of Partek Genomics Suite. All other graphs were plotted and statistical analyses were performed using GraphPad PRISM^®^ Version 5 software.

## Additional Information

**How to cite this article:** Lee, C. *et al*. Selective induction of alternatively spliced FynT isoform by TNF facilitates persistent inflammatory responses in astrocytes. *Sci. Rep.* **7**, 43651; doi: 10.1038/srep43651 (2017).

**Publisher's note:** Springer Nature remains neutral with regard to jurisdictional claims in published maps and institutional affiliations.

## Supplementary Material

Supplementary tables and figures
