# Supplementary material for: Selective induction of alternatively spliced FynT isoform by TNF facilitates persistent inflammatory responses in astrocytes
Source: Sci Rep. 2017 Mar 7;7:43651. doi: 10.1038/srep43651 (PMC5339870; doi:10.1038/srep43651)
Supplement: Supplementary tables and figures [file srep43651-s1.pdf]

**Selective induction of alternatively spliced FynT isoform by TNF facilitates persistent inflammatory responses in astrocytes**

Chingli Lee[1,2], Clara Y.B. Low[1], Siew Ying Wong[2], Mitchell K.P. Lai[2] and Michelle G.K. Tan[1,2*]

[1]Department of Clinical Translational Research, Singapore General Hospital, The Academia, Level 9, Discovery Tower, 20 College Road, Singapore 169856. [2]Department of Pharmacology, Yong Loo Lin School of Medicine, National University of Singapore, Level 5, CRC Building (MD11), 10 Medical Drive, Singapore 117597.

*Correspondence and requests for materials should be addressed to Michelle G.K Tan (email: michelle.tan.g.k@sgh.com.sg)

**Supplementary Table S1: Primary and secondary antibodies used in 'Selective induction of alternatively spliced FynT isoform by TNF facilitates persistent inflammatory responses in astrocytes'**

| Antibody Name (Catalog #) | Species | Dilution | Company |
|---|---|---|---|
| GFAP (GA5) (#3670)* | mouse mAb | 1:100 (IF) | Cell Signaling |
| Anti-NeuN (ABN78) | rabbit pAb | 1:100 (IF) | Millipore |
| Anti-NeuN, clone A60, Alexa Fluor 555-conjugate (MAB377A5)* | mouse mAb | 1:100 (IF) | Millipore |
| Custom-made FynT-specific* | rabbit pAb | 1:100 (IF) | GenScript |
| Anti-mouse IgG, Alexa Fluor 555-conjugate (#4408) | goat pAb | 1:1000 (IF) | Cell Signaling |
| Anti-rabbit IgG, Alexa Fluor 488 conjugate (#4412)* | goat pAb | 1:1000 (IF) | Cell Signaling |
| Anti-mouse IgG, Alexa Fluor 647-conjugate (A21235)* | goat pAb | 1:1000 (IF) | ThermoScientific |
| Total Fyn (#4023) | rabbit pAb | 1:1000 (WB) | Cell Signaling |
| Phospho-Src family (Tyr416) (#2101) | rabbit pAb | 1:1000 (WB) | Cell Signaling |
| PKCδ (D10E2) (#9616) | rabbit mAb | 1:1000 (WB) | Cell Signaling |
| Phospho-PKCδ (Tyr311) (#2055) | rabbit pAb | 1:1000 (WB) | Cell Signaling |
| IκBα (L35A5) (#4814) | mouse mAb | 1:1000 (WB) | Cell Signaling |
| NFκB p65 (D14E12)XP® (#8242) | rabbit mAb | 1:1000 (WB) | Cell Signaling |
| Phospho-NFκB p65 (Ser536) (93H1)(#3033) | rabbit mAb | 1:1000 (WB) | Cell Signaling |
| β-actin (13E5), HRP conjugate (#5125) | rabbit mAb | 1:3000 (WB) | Cell Signaling |
| Anti-mouse IgG, HRP-linked Ab (#7076) | goat pAb | 1:3000 (WB) | Cell Signaling |
| Anti-rabbit IgG, HRP-linked Ab (#7074) | goat pAb | 1:3000 (WB) | Cell Signaling |

(mAb= monoclonal antibody, pAb=polyclonal antibody, IF= Immunofluorescence staining, WB=Western blot analysis). *Antibodies used for triple-IF staining.

**Supplementary Table S2. Primers used in 'Selective induction of alternatively spliced FynT isoform by TNF facilitates persistent inflammatory responses in astrocytes'**

| Primer Name | Forward primer seq (5'-3') | Reverse primer seq (5'-3') | Accession no (product size) |
|---|---|---|---|
| FynB | CTGCTGCCGCCTAGTAGTTC | GTGTTTCCATTCCAGGTACC | NM_002037(168bp) |
| FynT | CATCGAGTTGTACCCCACAA | GTGTTTCCATTCCAGGTACC | NM_153047(136bp) |
| CCL2 | GCCTCCAGCATGAAAGTCTC | AGATCTCCTTGGCCACAATG | NM_002982(232bp) |
| CCL5 | CGCTGTCATCCTCATTGCTA | GAGCACTTGCCACTGGTGTA | NM_001278736(150bp) |
| CCL7 | CCTCCAACATGAAAGCCTCT | CCAGCCTCTGCTTAGGGATT | NM_006273(153bp) |
| CXCL8 (IL-8) | TAGCAAAATTGAGGCCAAGG | AAACCAAGGCACAGTGGAAC | NM_000584(227bp) |
| CXCL10 | AGGAACCTCCAGTCTCAGCA | CAAAATTGGCTTGCAGGAAT | NM_001565(192bp) |
| CXCL12 | TGAGAGCTCGCTTTGAGTGA | CACCTTGCCAACAGTTCTGA | NM_199168(192bp) |
| CSF2 (GM-CSF) | ATGTGAATGCCATCCAGGAG | AGGGCAGTGCTGCTTGTAGT | NM_000758(224bp) |
| CSF3 (G-CSF) | ACGAGGGTCAGGACTGTGAC | GTGACAGTGGAGGGGACACT | NM_000759(189bp) |
| IL1B (IL-1β) | AGCCAGGACAGTCAGCTCTC | AAGCGGTTGCTCATCAGAAT | NM_000576(171bp) |
| IL1RN (IL-1ra) | ACCAATATGCCTGACGAAGG | GTGACCAGGTTGTTGTGACG | NM_173841(209bp) |
| IL6 | GTCCACTGGGCACAGAACTT | CAAACTGCATAGCCACTTTCC | NM_000600(167bp) |
| IL23 | GTGGGACACATGGATCTAAGAGAA | TCAGACCCTGGTGGATCCTT | NM_016584(142bp) |
| LIF | CCCTGGTCCCTACTCAACAA | CTGGACCCTGACACCCTAAA | NM_001257135(234bp) |
| TNFR1 | GTGCCTACCCCAGATTGAGA | TGTCGATTTCCCACAAACAA | NM_001065(175bp) |
| PKCδ | GAAGAAGACCGGAGTTGCTG | GTCGATCAGGACCACATCCT | NM_006254(214bp) |
| 18S rRNA | CCTGCGGCTTAATTTGACTC | CGCTGAGCCAGTCAGTGTAG | M10098(310bp) |
| α-tubulin | ATGGAGCCCTGAATGTTGAC | ACATCTTTGGGAACCACGTC | K00558(250bp) |
| β-actin | ACTGGAACGGTGAAGGTGAC | AGAGAAGTGGGGTGGCTTTT | NM_001101(169bp) |
| GAPDH | TGACATCAAGAAGGTGGTGAAG | TTACTCCTTGGAGGCCATGTG | M33197(241bp) |
| Ms FynB | CTGCTGCCGCCTAGTAGTTC | GTATTTCCATTCCAGGTACC | NM_001122893(168bp) |
| Ms FynT | CATCAAGTTGTACCCCACAA | GTATTTCCATTCCAGGTACC | NM_008054(136bp) |
| Ms GFAP | TGAGGCAGAAGCTCCAAGAT | CACGTGGACCTGCTGTTG | NM_001131020(215bp) |
| Ms GAPDH | GGCATTGCTCTCAATGACAA | TGTGAGGGAGATGCTCAGTG | NM_008084(200bp) |
| Rat FynB | CTGCTGCCGTCTAGTAGTTC | GTGTTTCCATTCCAGGTACC | XM_006256520(168bp) |
| Rat FynT | CATCAAGTTGTACCCCACAA | GTGTTTCCATTCCAGGTACC | XM_006256521(136bp) |
| Rat GFAP | TGAGGCAGAAGCTCCAAGAT | CTCGAACTTCCTCCTCATGG | NM_017009(177bp) |
| Rat GLAST | ATCCAGGCCAACGAAACACT | CTTCATGTTTCCGATCACGA | NM_019225(177bp) |
| Rat NeuN | TTCCCACCACTCTCTTGTCC | ATCAGCAGCCGCATAGACTC | NM_001134498(168bp) |
| Rat PKCδ | ACTCATTTTCCGTGGACTGG | GTCCTTGGACTCCTTGGTGA | NM_133307(158bp) |
| Rat β-actin | ACTGGAACGGTGAAGGCGAC | TGCCGTGGATACTTGGAGTG | V01217(134bp) |
| Rat GAPDH | GGCATTGCTCTCAATGACAA | TGTGAGGGAGATGCTCAGTG | NM_017008(223bp) |
| Rat 18S rRNA | CCTGCGGCTTAATTTGACTC | CGCTGAGCCAGTTCAGTGTA | NR_046237(320bp) |
| *Rat Fyn | GGCCCAGTTTGAGACCCTTC | *GTGTTTCCATTCCAGGTACC | (228bp for FynB)<br>(219bp for FynT) |

(* indicate Fyn primer sets for fragment analysis using capillary electrophoresis, with 5' labeled 6-FAM on reverse primer)

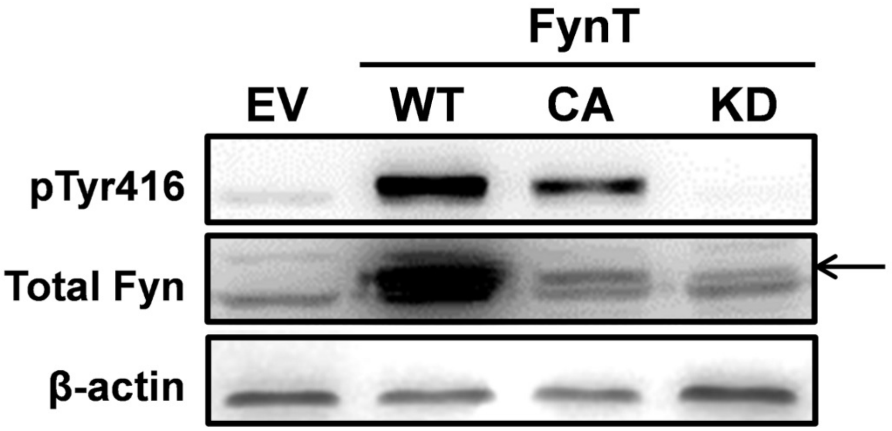


**Supplementary Figure S1. Characterization of iNHA FynT clones.** Representative iNHA clones stably expressing empty vector (EV), FynT wild-type (WT) and FynT mutants of constitutively active (CA) or kinase dead (KD) were detected for ectopic FynT expression (upper band, indicated by arrow) and endogenous Fyn (lower band) by anti-Fyn antibody. Autophosphorylated Fyn detected by anti-pTyr416 antibody indicated the presence of Fyn kinase activity. β-actin was served as an endogenous loading control.

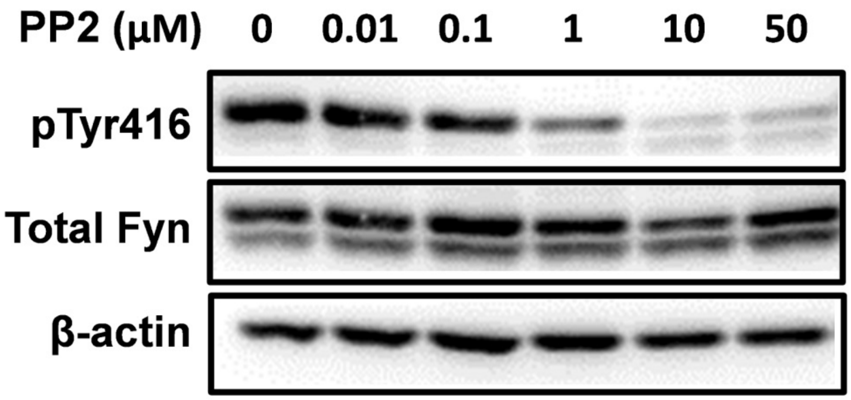


**Supplementary Figure S2. PP2 exhibited dose-dependent inhibition of Fyn tyrosine kinase activity in FynT-CA clone.** FynT-CA clone treated with different dose of PP2 were monitored using immunoblot analysis for ectopic FynT tyrosine kinase activity and total Fyn protein level using anti-pTyr416 and anti-Fyn antibodies, respectively. β-actin was used as a loading control.

**a**

**DsiRNA_FynT 1**

5′- GGAUUGGCUAAAGAUGCUUGGGAag-3′
3′-GACCUAACCGAUUUCUACGAACCCUUC-5′

**DsiRNA_FynT 2**

5′- ACUUAACUGUGAUUGCAUCGAGUtg-3′
3′-AUUGAAUUGACACUAACGUAGCUCAAC-5′

**DsiRNA_FynT 3**

5′- GUGUUUCGCUGAAGUGUGGCUUGgt-3′
3′-CCCACAAAGCGACUUCACACCGAACCA-5′

**DsiRNA_NC (Control Duplex)**

5′- CGUUAAUCGCGUAUAAUACGCGUat-3′
3′-CAGCAAUUAGCGCAUAUUAUGCGCAUA-5′

**b**

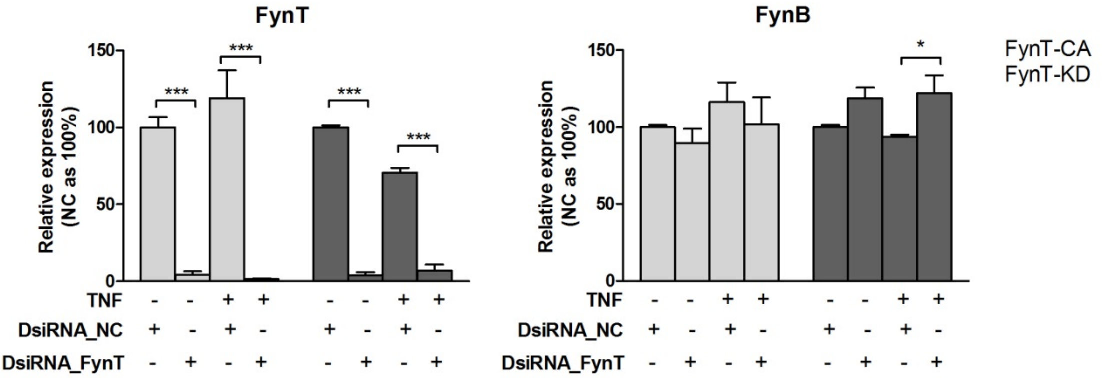


**Supplementary Figure S3. DsiRNA specific targeting FynT enable effective inhibition of ectopic FynT expression in FynT-CA and FynT-KD clone. (A)** RNA Duplex sequences were shown for three DsiRNAs specific targeting FynT (DsiRNA_FynT) and control duplex (DsiRNA_NC). Note that the DsiRNA is asymmetric 25/27-mer RNA duplex with a 3’ two-nucleotide overhang on the antisense strand and a blunt end modified with DNA bases (shown in lower case). (**B)** Real-time RT-PCR revealed that 30nM of pooled DsiRNA_FynT mixture was able to specific silencing the ectopic expression of FynT in both FynT-CA and FynT-KD clone in the presence or absence of TNF treatment. Relative expressions of FynT and FynB were normalized by geometric means of housekeeping genes and set the expression level in DsiRNA_NC transfection control without TNF treatment in each clone as 100%. Values are the means and ± SEM of five independent biological replicates. Two-way ANOVA with Bonferroni’s *post-hoc* test was conducted independently in each clone *(*** P≤ 0.001, * P≤ 0.05)*.

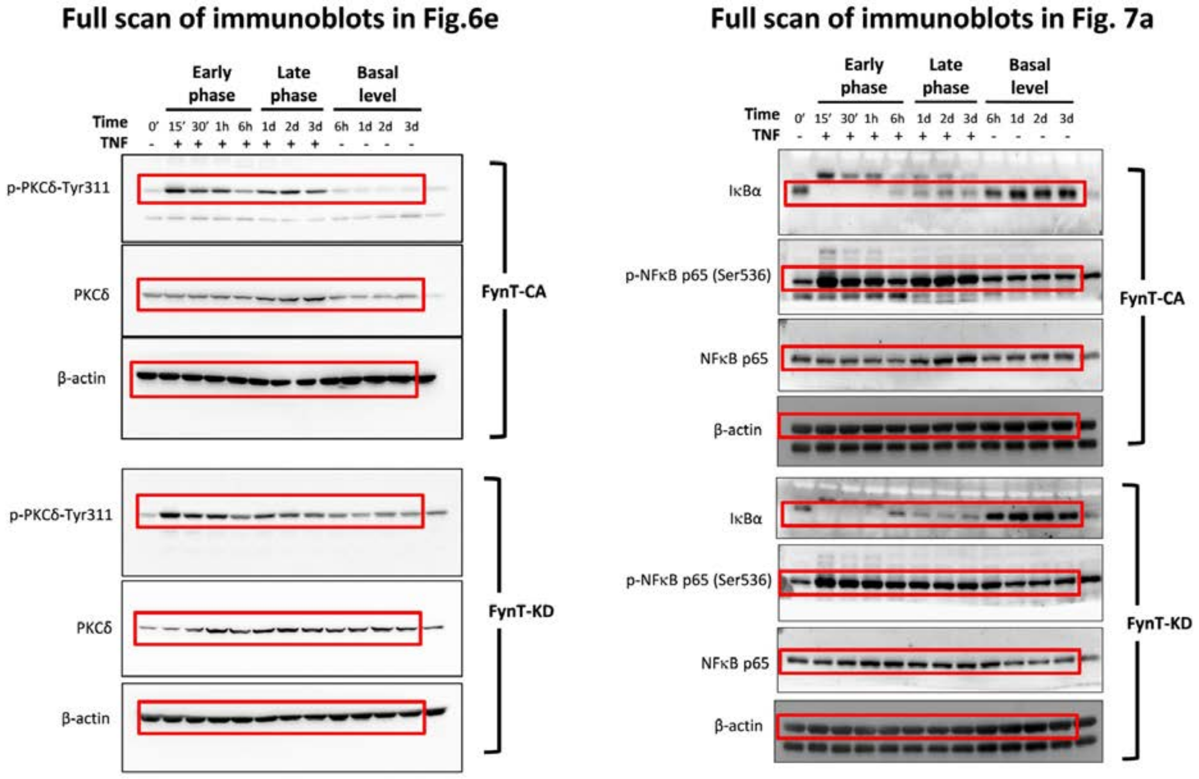


**Supplementary Figure S4. Full scans of the immunoblots presented in the paper.** The rectangles indicate the cropped images presented in the paper.
